# Association between GLP-1 receptor agonist use and substance use disorders among individuals with type 2 diabetes or obesity: a nested case-control study in the All of Us research program

**DOI:** 10.3389/fpsyt.2026.1766770

**Published:** 2026-03-10

**Authors:** Tadesse M. Abegaz, Muktar Ahmed, Akshaya Srikanth Bhagavathula, Gabriel Frietze

**Affiliations:** 1 School of Pharmacy, University of Texas at El Paso, El Paso, TX, United States; 2 College of Medicine and Public Health, Flinders University, Adelaide, SA, Australia; 3 Department of Public Health, College of Health and Human Services, North Dakota State University, Fargo, ND, United States

**Keywords:** alcohol use, All of Us cohort, cocaine dependence, drug repurposing, GLP-1 receptor agonists, nicotine dependence, opioid use, substance use disorders

## Abstract

**Objectives:**

The current study evaluated the association between glucagon-like peptide-1 receptor agonists (GLP-1 RAs) and substance use disorders (SUD) in type 2 diabetes and obesity.

**Methods:**

We conducted a retrospective nested case-control study using the All of Us Research Program data. Cases were defined as diabetes/obese individuals with a new diagnosis of alcohol use disorder (AUD), opioid use disorder (OUD), nicotine use disorder (NUD), or cocaine use disorder (CUD). Control participants were drawn from individuals with diabetes or obesity who had no documented history of SUD. Conditional logistic regression was performed to estimate the association between SUD and GLP-1 RA exposure.

**Results:**

The study included a total of 22,652 participants in the AUD group, 13,226 in the OUD group, 42,320 in the NUD group, and 9,296 in the CUD group; each group comprised both cases and matched control participants. GLP-1 RA use was associated with a 74% reduction in the odds of AUD (odds ratio [OR] = 0.26; 95% confidence interval [CI]: 0.20–0.34), a 69% reduction in the odds of OUD (*OR* = 0.31; 95% CI: 0.23–0.42), a 68% reduction in the odds of NUD (*OR* = 0.32; 95% CI: 0.27–0.39), a 75% reduction in the odds of CUD (*OR* = 0.25; 95% CI: 0.16–0.40), and 75% lower odds of any SUD compared with non-users participants (OR = 0.25, 95% CI 0.22–0.30).

**Conclusions:**

GLP-1 RA use was consistently associated with lower odds of developing SUDs among individuals with type 2 diabetes or obesity. These findings suggest potential for GLP-1 RAs to help mitigate SUD in these populations.

## 1 Introduction

Substance use disorders (SUDs) represent a major global public health challenge, contributing significantly to morbidity, premature mortality, and socioeconomic burdens (1). The World Health Organization estimates that over 270 million people worldwide are affected by SUDs, with annual cost exceeding $700 billion due to healthcare utilization, criminal justice involvement, and productivity losses (2). The annual medical cost associated with SUD in the United States exceeded $13 billion in 2017, highlighting the substantial economic burden of SUD on our healthcare system (3). While pharmacotherapies combined with behavioral interventions can be effective, relapse rates remain high, exceeding 50% within the first-year post treatment (4–6). Some behavioral strategies are limited in scalability, efficacy, poor long-term adherence, and accessibility issues, indicating the urgent need for novel, targeted interventions that can reduce SUD (7–10).

One promising strategy involves repurposing existing medications with well-established safety profiles (11). For example, glucagon-like peptide-1 receptor agonists (GLP-1 RAs s), originally developed for the treatment of type 2 diabetes and obesity, have recently emerged as potential candidates in the treatment of SUDs (12). Agents such as liraglutide and semaglutide, regulate metabolic functions by enhancing glucose-dependent insulin secretion, contributing to a suppression in appetite and weight loss (13). Studies suggested that GLP-1 receptors are located in the human brain—particularly within the hypothalamus, underline a central mechanism contributing to their effects on glucose metabolism and weight loss (14, 15). GLP-1 receptors are expressed in brain regions central to reward and reinforcement, including the ventral tegmental area and nucleus accumbens. Preclinical research indicated that that GLP-1 RAs attenuate dopamine release, consequently reducing cravings and compulsive drug-seeking behaviors (13, 16, 17). Animal studies have demonstrated that GLP-1 RAs reduce the self-administration of alcohol, opioid, nicotine, and cocaine that effects are believed to be mediated through interactions with the mesolimbic dopamine system. For example, liraglutide (0.1 mg/kg) significantly reduced voluntary alcohol consumption in male rats using a two-bottle choice paradigm; even lower doses (0.05 mg/kg) were effective in alcohol-preferring strains (18).

Despite promising findings in animal studies, human studies remain limited. Observational data among patients with alcohol use disorder (AUD) and comorbid diabetes or obesity suggests that GLP-1 RAs use may be associated with reduced risk of AUD-related hospitalization (19). Small-scale clinical trials and cohort studies have also reported decreased substance cravings and relapse rates among individuals treated with GLP-1 RAs (20–24). However, these studies are constrained by small sample sizes, short follow-up periods, and limited generalizability across diverse populations.

Notably, the real-world impact of GLP-1 RA on multiple co-occurrence of SUD has not been systematically investigated. The current study assessed whether the use of GLP-1 RAs is associated with a reduced risk of subsequent SUD diagnosis using a nested case-control design. We hypothesized that individuals who are using GLP-1 RAs would have lower odds of developing SUDs compared to non-users. By leveraging the All of Us Research Program data, a nationally representative longitudinal health data from a diverse population, our findings provide new insights into the potential of GLP-1 RAs as a novel therapeutic strategy for addiction management and lay the groundwork for future clinical and translational research.

## 2 Materials and methods

### 2.1 Data source

Data was obtained from the AoU Research Program, a National Institutes of Health-funded initiative designed to collect comprehensive health data from diverse participants to advance precision medicine. The dataset includes longitudinal electronic health records, prescription medication records, sociodemographic information, and participant-reported health surveys. Data were accessed through the secured All of Us Researcher Workbench, a cloud-based platform compliant with federal privacy and security standards (25). For this study, we used the most recent All of Us Controlled Tier Dataset, version 8 (v8), which was released in February 2025.

### 2.2 Study design and population

We conducted a retrospective nested case–control study within a well-defined cohort of adults enrolled in the All of Us Research Program to evaluate the association between GLP-1 RAs exposure and incident SUD diagnoses. A nested case–control design is efficient for evaluating relatively uncommon outcomes (I.e.,substance use disorder) within a large cohort and supports clear temporality between exposure and outcome through index-date anchoring and matching.

The study population included adults aged 18 years and older who were enrolled in the All of Us Research Program. To ensure temporality between GLP-1 RA use and SUD onset, we established the observation window from January 1, 2005 (the year of initial GLP-1 RA approval) to February 24, 2025, based on the most recent data available from the AoU dataset.

### 2.3 Cases and control definition

Cases were defined as individuals with a new (incident) diagnosis of SUD, including AUD, opioid use disorder (OUD), nicotine use disorder (NUD), or cocaine use disorder (CUD). The SUD cases in our study were identified using standardized clinical condition codes available in the All of Us Controlled Tier dataset. The All of Us Research Program uses the Common Data Model, in which Systematized Nomenclature of Medicine serves (SNOMED) as the standard vocabulary for conditions. The All of Us research program systematically maps the international code of diseases to standardized SNOMED concepts. Therefore, the SNOMED concepts used in our study directly represent ICD-derived diagnoses of AUD, OUD, NUD, and CUD. For this study, any SUD was operationalized as the presence of at least one diagnosis of AUD, OUD, NUD, or CUD. Participants were classified as having “any SUD” if they met criteria for one or more of these individual conditions. The index date was defined as the date of the first documented SUD diagnosis during the observation period. To ascertain incident cases, individuals with any prior SUD diagnosis before the observation window were excluded. In addition, participants with missing outcome variables, under 18 years old, and those with no history of diabetes or/and obesity were excluded. Controls were randomly selected from the All of Us cohort among participants with no documented SUD diagnosis throughout their follow-up. Each case was matched 1:1 to a control using propensity score matching with a nearest-neighbor algorithm without replacement. The following covariates: age, sex, race/ethnicity, type 2 diabetes or obesity status, and type of oral hypoglycemic agents were during matching. Covariate balance before and after matching was assessed using standardized mean differences, with values <0.1 considered indicative of acceptable balance.

### 2.4 Exposure definition

The primary exposure was GLP-1 RA use, defined as any documented prescription fill for a GLP-1 RA (liraglutide, semaglutide, exenatide, and dulaglutide) at least 90 days prior to the index date, based on prescription records in the electronic health record. This 90-day lag was implemented to account for the time required for GLP-1 RAs to exert potential neuromodulatory effects and to minimize reverse causality. Preclinical studies demonstrate that sustained GLP-1 receptor activation alters mesolimbic dopamine signaling over a period of time, and emerging studies suggest that behavioral and craving-related effects require continued exposure (26, 27).

The start date of prescription were identified using the Observational Medical Outcomes Partnership Common Data Model (CDM). Exposure was coded as a binary variable (1 = exposed, 0 = unexposed). Non-GLP-1RA users (unexposed) were individuals with no record of GLP-1RA prescriptions before the index date.

### 2.5 Statistical analysis and sensitivity analyses

Descriptive analyses were conducted to examine baseline differences between case and control groups. Categorical variables are reported as frequencies and percentages, while continuous variables are summarized using means and standard deviations. As all eligible cases from the database were included and the sample size was inherently determined by disease incidence, no formal sample size or power calculation was carried out.

The association between GLP-1 RA exposure and the odds of SUD was estimated to use conditional logistic regression, which accounted for the nested case control design. The primary outcome was the odds of each SUD subtype (AUD, OUD, NUD, or CUD). Subgroup analyses stratified by the type of GLP-1 RA were performed. In addition, sensitivity analyses were conducted based on obesity and gender difference. Results were reported as odds ratios (ORs) and corresponding 95% confidence intervals (CIs). Statistical significance was defined as a two-sided p-value <0.05. Model fit was assessed using the Hosmer-Lemeshow goodness-of-fit test. Missing data for covariates (<5% for most variables) were handled using multiple imputations by chained equations. All analyses were performed using R (version 4.5.0) within the AoU Researcher Workbench environment. We followed the Strengthening the Reporting of Observational studies in Epidemiology guidelines to ensure transparent and comprehensive reporting of this observational study (28).

## 3 Results

### 3.1 Baseline characteristics of participants

Among 142,349 participants with type 2 diabetes or obesity in the All of Us Research Program, individuals were classified into four SUD cohorts: AUD, OUD, NUD, and CUD. After identifying cases, final analytic sample sizes were 22,652 for AUD, 13,226 for OUD, 22,320 for NUD, and 9,296 for CUD.

The AUD cohort included 11,326 cases and 11,326 controls (n=22,652) participants. The mean age in the AUD cohort was similar between cases and controls (55.1 years; standard deviation [SD] = 15). Males represented 56.1% of cases and 53.9% of controls, while females accounted for 41.6% and 45.6%, respectively. White participants comprised 43.4% of cases and 46.3% of controls, and Black participants 27.3% and 25.4%, respectively. Approximately 79% of participants in both groups identified as non-Hispanic, and around 17-18% as Hispanic. GLP-1 RA use was similar between groups (0.7% in cases vs. 0.9% in controls) (**Table 1**).

**Table 1:** Baseline characteristics of participants in case and control groups across each SUD cohort.

Variables	AUD(n=22,652)		OUD (n=13,226)		NUD (n=42,320)		CUD (n=9,296)	
	Case	Control	Case	Control	Case	Control	Case	Control
	(n=11326)	(n=11326)	(n= 6,613)	(n= 6,613)	(n=21160)	(n=21160)	(n=4648)	(n=4648)
Age(mean/SD)	55.1 (15)	55.1 (15)	52.2 (13)	52.6 (14.6)	54 (14.5)	53.8 (15.8)	53.1 (11)	52.8 (13.9)
Duration on GLP-RA (days)	725.8 (92.9)	725.8 (92.9)	717.9 (95.2)	717.9 (95.2)	726.8 (92.7)	726.8 (92.7)	717.8 (93.2)	717.8 (93.2)
Gender								
Male	6352 (56.1%)	6096 (53.9%)	2820 (42.6%)	2713 (41%)	9205 (43.5%)	8631 (40.8%)	2476 (53.3%)	2302 (49.5%)
Female	4705 (41.6%)	5159 (45.6%)	3637 (55%)	3833 (58%)	11513 (54.4%)	12331 (58.3%)	2046 (44%)	2310 (49.7%)
Others	256 (2.3%)	58 (0.5%)	156 (2.4%)	67 (1%)	442 (2.1%)	198 (0.9%)	126 (2.7%)	36 (0.8%)
Race								
White	4909 (43.4%)	5235 (46.3%)	3008 (45.5%)	2966 (44.9%)	9615 (45.4%)	9615 (45.4%)	942 (20.3%)	1454 (31.3%)
Black	3090 (27.3%)	2869 (25.4%)	1745 (26.4%)	1847 (27.9%)	6033 (28.5%)	5375 (25.4%)	2440 (52.5%)	1672 (36%)
Asian	55 (0.5%)	119 (1.1%)	33 (0.5%)	58 (0.9%)	137 (0.6%)	274 (1.3%)	16 (0.3%)	48 (1%)
Others	3090 (27.3%)	3090 (27.3%)	1827 (27.6%)	1742 (26.3%)	5763 (27.2%)	5896 (27.9%)	1250 (26.9%)	1474 (31.7%)
Ethnicity								
Non-Hispanic	8957 (79.2%)	9002 (79.6%)	5276 (79.8%)	5313 (80.3%)	16775 (79.3%)	16721 (79%)	3757 (80.8%)	3796 (81.7%)
Hispanic	2356 (20.9%)	2311 (20.5%)	1337 (20.2%)	1300 (19.7%)	4385 (20.7%)	4439 (21%)	891 (19.2%)	852 (18.3%)
Education								
No high school diploma	4305 (38%)	4699 (41.5%)	2623 (39.7%)	2851 (43.1%)	8405 (39.7%)	9509 (44.9%)	1723 (37.1%)	2007 (43.2%)
High school diploma	4676 (41.3%)	3890 (34.3%)	3019 (45.7%)	2638 (39.9%)	8609 (40.7%)	6740 (31.9%)	2538 (54.6%)	2038 (43.8%)
College and above	2345 (20.7%)	2737 (24.2%)	971 (14.7%)	1124 (17%)	4146 (19.6%)	4911 (23.2%)	387 (8.3%)	387 (8.3%)
Employment								
Employed	2292 (20.2%)	2263 (20%)	700 (10.6%)	651 (9.8%)	5091 (24.1%)	5193 (24.5%)	483 (10.4%)	420 (9%)
Unemployed	9034 (79.8%)	9063 (80%)	5913 (89.4%)	5962 (90.2%)	16069 (75.9%)	15967 (75.5%)	4165 (89.6%)	8393 (90.3%)
Annual Income								
< 25k	4743 (41.9%)	4531 (40%)	3311 (50.1%)	3197 (48.3%)	8305 (39.2%)	7941 (37.5%)	2773 (59.7%)	2662 (57.3%)
25k-50k	1608 (14.2%)	1830 (16.2%)	817 (12.4%)	922 (13.9%)	3339 (15.8%)	3668 (17.3%)	430 (9.3%)	591 (12.7%)
50k-100k	1416 (12.5%)	1579 (13.9%)	566 (8.6%)	673 (10.2%)	2805 (13.3%)	3180 (15%)	171 (3.7%)	334 (7.2%)
100k-200k	663 (5.9%)	852 (7.5%)	173 (2.6%)	252 (3.8%)	1293 (6.1%)	1571 (7.4%)	48 (1%)	134 (2.9%)
>200k	2896 (25.6%)	5430 (24%)	1746 (26.4%)	1569 (23.7%)	5418 (25.6%)	4800 (22.7%)	1226 (26.4%)	927 (19.9%)
Health Insurance								
Insured	10261 (90.6%)	10326 (91.2%)	6085 (92%)	6105 (92.3%)	19354 (91.5%)	19470 (92%)	4073 (87.6%)	4081 (87.8%)
Not insured	1000 (8.8%)	1000 (8.8%)	528 (8%)	508 (7.7%)	1806 (8.5%)	1690 (8%)	575 (12.4%)	567 (12.2%)
Marital Status								
Married	3012 (26.6%)	2994 (26.4%)	1476 (22.3%)	1432 (21.7%)	6296 (29.8%)	6291 (29.7%)	596 (12.8%)	514 (11.1%)
Unmarried	8314 (73.4%)	8332 (73.6%)	5137 (77.7%)	5181 (78.3%)	14864 (70.2%)	14869 (70.3%)	4052 (87.2%)	4134 (88.9%)
Medications								
GLP-1 Agonist	74 (0.7%)	287(2.5%)	61 (0.9%)	184 (2.4%)	174 (0.8%)	503(2.4%)	25 (0.5%)	90(2%)
Metformin	3130 (27.7%)	2993 (26.5%)	1689 (25.5%)	1628 (24.6%)	5792 (27.4%)	5570 (26.3%)	1405 (30.2%)	1385 (29.8%)
Sulfonylureas	1106 (9.8%)	1004 (8.9%)	594 (9%)	556 (8.4%)	2061 (9.7%)	1979 (9.4%)	462 (9.9%)	447 (9.6%)
Insulin	4270 (37.7%)	4108 (36.3%)	2956 (44.7%)	2877 (43.5%)	8041 (38%)	7688 (36.3%)	2040 (43.9%)	2029 (43.7%)
SGLT2i	784 (6.9%)	713 (6.3%)	363 (5.5%)	342 (5.2%)	1360 (6.4%)	1297 (6.1%)	331 (7.1%)	285 (6.1%)
DPP4i	518 (4.6%)	483 (4.3%)	278 (4.2%)	262 (4%)	5235 (24.7%)	5157 (24.4%)	1101 (23.7%)	1029 (22.1%)
Naltrexone	851 (7.5%)	851 (7.5%)	447 (6.8%)	389 (5.9%)	843 (4%)	632 (3%)	454 (9.8%)	377 (8.1%)
Statin	5639 (24.9%)	2755 (24.4%)	3028 (45.8%)	3009 (45.5%)	10635 (50.3%)	10300 (48.7%)	2530 (54.4%)	2383 (51.3%)
Comorbidity								
DM	6343 (56.1%)	6101 (53.9%)	3834 (58%)	3806 (57.6%)	11793 (55.7%)	11547 (54.6%)	3363 (72.4%)	3393 (73%)
Obesity	8470 (74.9%)	8739 (77.2%)	5171 (78.2%)	5255 (79.5%)	16490 (77.9%)	16675 (78.8%)	2852 (61.4%)	2817 (60.6%)

AUD, Alcohol use disorder, CUD, cocaine use disorder, NUD, Nicotine use disorder, OUD, Opioid use disorder. To comply with the All of Us data dissemination policy and protect participant privacy, variables with fewer than 20 participants were not released or published.

The OUD cohort, on the other hand, comprised of 6,613 cases and 6,613 controls (n = 13,226). The mean age was comparable (52.2 vs. 52.6 years) across cases and controls in the OUD cohort. Females accounted 55% of cases and 58% of controls. Racial and ethnic profiles were also comparable: approximately 45% of participants were White in both groups, and ~20% identified as Hispanic. GLP-1 RA use was higher among controls (2.5%) than cases (0.9%) (**Table 1**).

The NUD cohort included 21,160 cases and 21,160 controls (n=42,320). Mean age in the NUD cohort was similar between cases and controls (54.0 vs. 53.8 years). Slightly more females were observed in the control group (58.3%) compared to cases (54.4%). Racial distributions were balanced, with non-Hispanic Whites representing 45.4% of both groups and Black individuals 28.5% vs. 25.4% (cases vs. controls). Hispanic ethnicity represented (~21% in both groups). GLP-1 RA use was more frequent among controls (2.4%) than cases (0.8%) (**Table 1**).

The CUD cohort included 4, 648, cases and 4, 648 controls (n=9296) with mean age (53.1 vs. 52.8 years). A higher proportion of males were observed among cases (53.3%) compared to controls (49.5%). Black individuals constituted 52.5% of cases and 36.0% of controls, while White participants made up to 31.3% and 20.3% respectively (**Table 1**).

We also evaluated the co-occurrence of different substance use problems. Among 142,349 participants with type 2 diabetes or obesity in the All of Us Research Program, we assessed co-occurrence across four SUD phenotypes—AUD, CUD, OUD, and NUD. Overall, 27,823 participants had exactly one SUD diagnosis (19.5%), 8,332 had two SUD diagnoses (5.9%), and 3,028 had three SUD diagnoses (2.1%). A total of 1,133 participants had all four SUD diagnoses (0.8%).

We further examined pairwise co-occurrence among AUD, OUD, NUD, and CUD. Among the study population, AUD co-occurred most frequently with NUD, affecting 7,769 participants (5.5%). Co-occurrence of AUD and CUD was observed in 3,022 participants (2.1%), while AUD and OUD co-occurred in 2,535 participants (1.8%). These findings highlight substantial heterogeneity in SUD comorbidity patterns, with alcohol–nicotine related-disorders representing the most prevalent pairing in this population (**Supplementary Figure 1**).

### 3.2 Association between GLP-1RA exposure and alcohol use disorder

**Figure 1:**
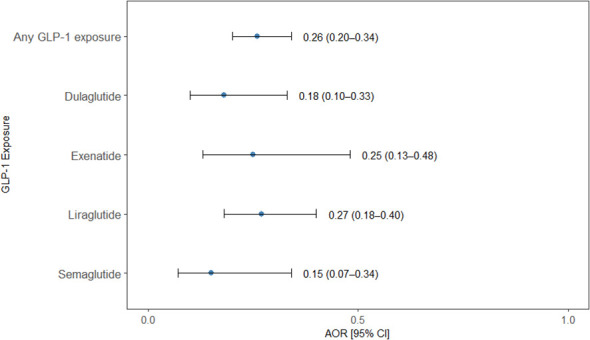
Association between GLP-1 RAs exposure and alcohol use disorder. Forest plot showing adjusted odds ratios (AORs) and 95% confidence intervals (CIs) for the association between any GLP-1RA exposure and individual GLP-1 RAs (dulaglutide, exenatide, liraglutide, and semaglutide) with the odds of AUD in a nested case–control analysis. Points represent AORs and horizontal lines indicate 95% CIs. All GLP-1RA exposures were associated with significantly lower odds of AUD compared with non-exposure.

In this nested case-control analysis, exposure to GLP-1 RA was consistently associated with significantly reduced odds of an AUD diagnosis. Overall, any exposure to GLP-1 RA was associated with 74% lower odds of AUD (OR = 0.26; 95% CI: 0.20-0.34; *p* = 0.0001). When analyzed by specific agents, all GLP-1 RAs demonstrated statistically significant protective associations. The strongest association was observed with semaglutide, which was associated with an 85% reduction in odds of AUD (OR = 0.15; 95% CI: 0.07–0.34; *p* = 0.0001), followed by dulaglutide (OR = 0.18; 95% CI: 0.10-0.33, *p* = 0.001), exenatide (OR = 0.25; 95% CI: 0.13-0.48, *p* = 0.0001), and liraglutide (OR = 0.27; 95% CI: 0.18–0.40, *p* = 0.0001) (**Figure 1**).

### 3.3 Association between GLP-1RA exposure and opioid use disorder

**Figure 2:**
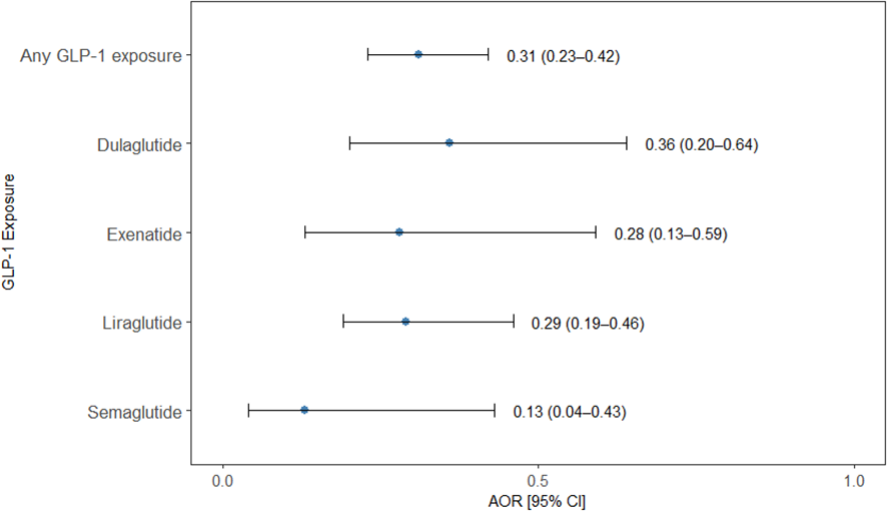
Association between GLP-1 RAs agonist exposure and opioid use disorder. Forest plot displaying adjusted odds ratios (AORs) with 95% confidence intervals (CIs) for the association between exposure to any GLP-1RA and individual GLP-1RAs (dulaglutide, exenatide, liraglutide, and semaglutide) and the odds of an OUD diagnosis in a nested case–control analysis. Points represent AORs and horizontal lines indicate 95% CIs.

Exposure to any GLP-1 RAs was significantly associated with reduced odds of OUD diagnosis. Overall, GLP-1 RA exposure was associated with 69% lower odds of OUD (OR = 0.31 (95% CI: 0.23-0.42, *p* = 0.0001). When examined individually, each GLP-1 RA demonstrated significant protective associations. Semaglutide was associated with the largest reduction in odds of OUD (OR = 0.13; 95% CI: 0.04-0.43; *p* = 0.0009), reflecting an 87% decrease in risk. Exenatide use was associated with a 72% reduction (OR = 0.28, 95% CI: 0.13-0.59, *p* = 0.0008), and liraglutide with a 71% reduction (OR = 0.29, 95% CI: 0.19-0.46, *p* = 0.0001). Dulaglutide use was associated with a 64% lower risk of OUD (OR = 0.36, 95% CI: 0.20-0.64, *p* = 0.0006) (**Figure 2**).

### 3.4 Association between GLP-1RA exposure and nicotine use disorder

**Figure 3:**
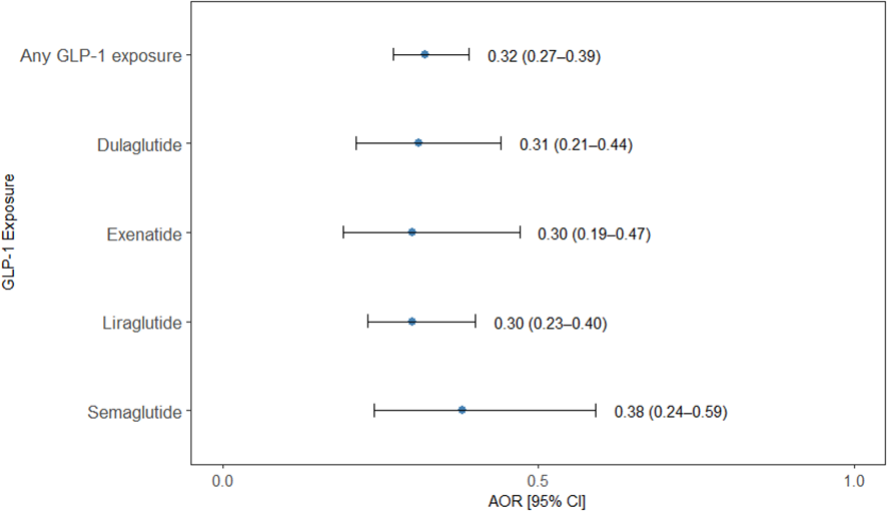
Association between GLP-1 RAs exposure and nicotine use disorder. Forest plot presenting adjusted odds ratios (AORs) with 95% confidence intervals (CIs) for the association between exposure to any GLP-1RA and individual GLP-1RA agents (dulaglutide, exenatide, liraglutide, and semaglutide) and the odds of a nicotine use disorder diagnosis in a nested case–control analysis. Points denote AORs and horizontal lines represent 95% CIs. Exposure to any GLP-1RA and to each individual agent was associated with significantly reduced odds of NUD compared with non-exposure.

Use of GLP-1 RAs was significantly associated with a reduced odd of NUD. Individuals exposed to any GLP-1 RA had 68% lower odds of NUD compared to unexposed individuals (odds ratio OR = 0.32; 95% CI: 0.27–0.39; *p* = 0.001). All four individual GLP-1 RAs demonstrated significant protective associations. Liraglutide was associated with the greatest reduction (OR = 0.30; 95% CI: 0.23-0.40; *p* < 0.001), followed by Exenatide (OR = 0.30; 95% CI: 0.19–0.47; *p* = 0.001), Dulaglutide (OR = 0.31; 95% CI: 0.21–0.44; *p* = 0.01), and semaglutide (OR = 0.38; 95% CI: 0.24-0.59; *p* = 0.001) (**Figure 3**).

### 3.5 Association between GLP-1RA exposure and cocaine use disorder

**Figure 4:**
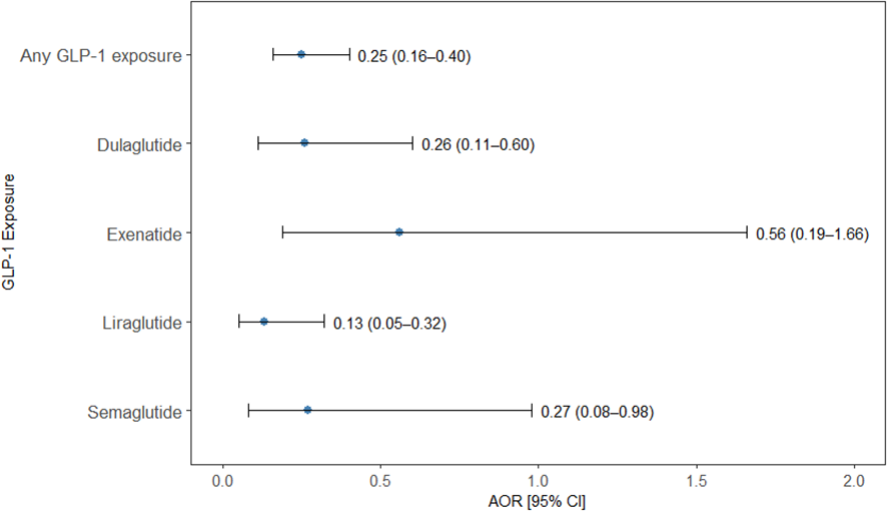
Association between GLP-1 RAs exposure and cocaine use disorder. Forest plot illustrating adjusted odds ratios (AORs) and 95% confidence intervals (CIs) for the association between exposure to any GLP-1RA and individual GLP-1RA agents (dulaglutide, exenatide, liraglutide, and semaglutide) and the odds of a cocaine use disorder diagnosis in a nested case–control analysis. Points indicate AORs and horizontal lines denote 95% CIs. Overall GLP-1RA exposure and most individual agents were associated with significantly reduced odds of CUD, with liraglutide showing the strongest protective association, while the association for exenatide did not reach statistical significance.

Exposure to GLP-1 RA was significantly associated with a reduced odds of CUD (OR = 0.25, 95%CI: 0.16–0.40, p = 0.0001). When analyzed by individual agents, liraglutide demonstrated the strongest protective association (OR = 0.13, 95% CI: 0.05–0.32, p = 0.0001), followed by dulaglutide (OR = 0.26, 95% CI: 0.11-0.60, p = 0.01) and semaglutide (OR = 0.27, 95% CI: 0.08–0.98, p = 0.046), all of which were statistically significant. Although exenatide was associated with a reduced odd (OR = 0.56), the finding was not statistically significant (95% CI: 0.19-1.66, p = 0.292) (**Figure 4**).

### 3.6 Association between GLP-1RA exposure and any substance use disorder

**Figure 5:**
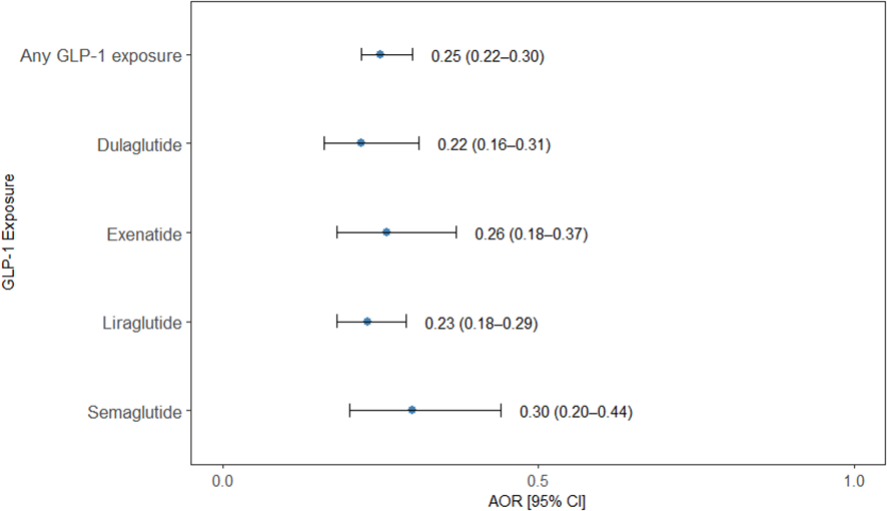
Association between GLP-1 receptor agonist exposure and the odds of any substance use disorder. Forest plot showing adjusted odds ratios (AORs) with 95% confidence intervals (CIs) for the association between exposure to any GLP-1RA and individual GLP-1RA agents (dulaglutide, exenatide, liraglutide, and semaglutide) and the odds of developing any substance use disorder in a nested case–control analysis. Points represent AORs and horizontal lines indicate 95% CIs. Exposure to any GLP-1RA and to each individual agent was associated with significantly lower odds of any SUD compared with non-exposure, with all confidence intervals excluding the null value.

Exposure to GLP-1 RA was associated with significantly lower odds of developing any substance use disorder. In our analysis, participants with any GLP-1RA exposure had 75% lower odds of any SUD compared with non-exposed participants (OR = 0.25, 95% CI 0.22–0.30). Estimates for individual agents were consistent with the overall finding: dulaglutide (OR = 0.22, 95% CI 0.16–0.31), exenatide (OR = 0.26, 95% CI 0.18–0.37), liraglutide (OR = 0.23, 95% CI 0.18–0.29), and semaglutide (OR = 0.30, 95% CI 0.20–0.44). All confidence intervals indicated statistically significant associations (**Figure 5**). Complete numerical estimates for **Figures 1**–**5**, including adjusted odds ratios, 95% confidence intervals, and p-values, are provided in **Supplementary Table 1**.

### 3.7 Stratified analyses by sex and obesity status

We conducted stratified analyses to evaluate whether the association between GLP-1RA exposure and the odds of substance use disorders differed by sex. Across all SUD outcomes and individual GLP-1RA agents, the direction and magnitude of the associations were generally comparable between females and males. No meaningful differences were observed between sex-specific estimates, providing no evidence of effect modification by sex (**Supplementary Figures 2**, **3**).

In addition, we performed stratified analyses by obesity status among participants with diabetes to assess whether obesity modified the association between GLP-1RA exposure and SUD risk. Across all SUD outcomes and individual GLP-1RA agents, effect estimates were largely consistent between participants with and without obesity. Overall, these subgroup analyses did not reveal meaningful heterogeneity in associations by obesity status, indicating no evidence of effect modification by obesity (**Supplementary Figures 4**, **5**).

## 4 Discussion

Several important findings emerged in this large, nested case-control study across different SUD cohorts. Exposure to GLP-1 RAs was consistently associated with significantly reduced odds of SUD cases. These findings suggest that GLP-1 RAs may exert behavioral-modifying effects that extend beyond glycemic control and weight loss.

The strongest associations were observed with semaglutide, which showed the most robust protective effect across all SUD categories, including an 85% reduction in AUD odds and an 87% reduction in OUD odds. Other GLP-1 RAs, including dulaglutide, liraglutide, and exenatide, also demonstrated statistically significant reductions in risk. Although all evaluated GLP-1RAs were associated with lower odds of SUD outcomes, the magnitude of association varied across some agents. These differences should be interpreted cautiously. In real-world data, GLP-1RAs differ in clinical indications, uptake over time, adherence rate, typical treatment duration, dose escalation, and patient selection, all of which can introduce residual confounding and channeling bias (29, 30). Beyond these real-world considerations, pharmacologic heterogeneity is biologically plausible given differences in molecular structure, receptor signaling profiles, half-life, and potency, which may influence downstream central effects (31).

Notably, the observed reductions in the odds of substance use disorders, including a 74% decrease in the odds of AUD, a 69% reduction for OUD, a 68% reduction for NUD, and a 75% reduction for CUD, among GLP-1 RA users were consistent with emerging preclinical and clinical studies showing that GLP-1 RA modulates reward pathways in the brain to reduce SUD (17, 23). Animal models have demonstrated that GLP-1 RAs reduce drug-seeking behavior and attenuate conditioned place preference for substances such as alcohol, nicotine, and cocaine (32). While the exact mechanisms remain to be fully elucidated, proposed pathways include modulation of dopamine release, suppression of neuroinflammation, and attenuation of stress-related neural circuits (27). These neurobiological effects may underline the observed reduction in SUD risk among GLP-1 RA users in our study. In addition, the expression of GLP-1 receptor in the hypothalamus regions could suggest that GLP-1RA treatment can alter central responses in humans (26, 33). Together with translational evidence, these data support the hypothesis that GLP-1 signaling can influence mesolimbic reward pathways, potentially through modulation of dopaminergic tone and downstream reinforcement learning processes (34). In the context of SUD, such mechanisms could plausibly reduce craving and compulsive drug-seeking, aligning with preclinical work showing attenuation of substance-related reinforcement and with emerging clinical data suggesting reduced alcohol-related outcomes among GLP-1RA users (18, 35). While our study does not establish causality or delineate mechanism, the consistency of associations across multiple SUD categories aligns with a shared neurobehavioral pathway influenced by GLP-1 receptor activation. However, the human evidence remains heterogeneous, and mixed findings may occur depending on study design, outcome definitions, and duration of exposure; therefore, additional randomized trials and well-controlled comparative effectiveness studies are needed.

### 4.1 Strengths and limitations of the study

This study has several notable strengths. First, we leveraged the All of Us Research Program, a large, diverse, dataset with longitudinal electronic health record data, improving the robustness of the findings to real-world clinical populations with diabetes and/or obesity. Second, the nested case–control design with propensity matching enabled efficient evaluation of multiple relatively uncommon outcomes while strengthening internal validity and supporting a clear temporal relationship between GLP-1RA exposure and SUD diagnoses. Third, we examined a broad spectrum of clinically relevant SUD outcomes including AUD, OUD, NUD, and CUD, allowing assessment of consistency across substances and supporting the possibility of a class effect. Fourth, the large sample sizes across the outcome cohorts provided adequate precision of effect estimates, and agent-specific analyses helped clarify whether associations were consistent across different GLP-1RA medications. Finally, we conducted subgroup analyses by sex and obesity status, demonstrating generally consistent associations across strata, and we implemented an exposure lag period to reduce potential reverse causality, collectively strengthening the credibility of the observed associations.

Nevertheless, the following limitations should be considered when interpreting these findings. First, the study population was limited to individuals with diabetes or obesity, which may restrict the generalizability of results to broader populations. Second, the observational nature of the study precludes causal inference, and residual confounding is possible. Third, although prescription start dates and diagnosis dates were identified using the Common Data Model, the temporal relationship between GLP-1 RA initiation and the onset of SUDs may not be established with complete certainty. In the Common Data Model framework, the diagnosis date reflects the time a diagnosis is first recorded in the medical record, which may not represent the true onset of symptoms. Finally, medication exposure was inferred from prescription records and may not reflect actual adherence.

### 4.2 Future directions

Future research should focus on confirming these findings in randomized controlled trials (RCTs) to establish causality and evaluate the effectiveness of GLP-1 RAs as therapeutic agents for SUDs. Mechanistic studies are also needed to further explore how GLP-1 RAs influence neural circuits implicated in reward, and reinforcement. Additionally, expanding the investigation to non-diabetic and non-obese populations will be critical for determining the broader applicability of these findings. Longitudinal studies tracking adherence, and duration of use, can provide valuable insights into sustained effects. Finally, future research should explore how GLP-1 RAs could be integrated into current clinical pathways for addiction care, especially in settings where diabetes, obesity, and SUD frequently co-occur.

### 4.3 Conclusion

In conclusion, our findings provide observational evidence that GLP-1 RA are associated with lower odds of developing multiple types of substance use disorders among individuals with diabetes or obesity. These results have implications for potentially repurposing GLP-1 RAs as novel therapeutic agents in addiction medicine. While further interventional research is necessary, the current study adds to the growing evidence on the neurobehavioral benefits of GLP-1 RAs and supports their dual utility in managing both metabolic and psychiatric comorbidities.

## Data Availability

The original contributions presented in the study are included in the article/**Supplementary Material**. Further inquiries can be directed to the corresponding author.

## References

[1] GBD 2019 Diseases and Injuries Collaborators . Global burden of 369 diseases and injuries in 204 countries and territories, 1990-2019: a systematic analysis for the Global Burden of Disease Study 2019. The Lancet. (2020) 396:1204–22. doi: 10.1016/S0140-6736(20)30925-9, PMID: 33069326 PMC7567026

[2] World Health Organisation . Global status report on alcohol and health and treatment of substance use disorders. Geneva, Switzerland: World Health Organization (2024).

[3] Peterson C Li M Xu L Mikosz CA Luo F . Assessment of annual cost of substance use disorder in US hospitals. JAMA Network Open. (2021) 4:e210242–e. doi: 10.1001/jamanetworkopen.2021.0242, PMID: 33666661 PMC7936257

[4] Suwanchatchai C Buaphan S Khuancharee K . Determinants and prevalence of relapse among patients with substance use disorder in a rural population: A retrospective observational study. J Subst Use Addict Treat. (2024) 157:209244. doi: 10.1016/j.josat.2023.209244, PMID: 38056631

[5] Jhanjee S . Evidence based psychosocial interventions in substance use. Indian J psychol Med. (2014) 36:112–8. doi: 10.4103/0253-7176.130960, PMID: 24860208 PMC4031575

[6] Dowell D Ragan KR Jones CM Baldwin GT Chou R . CDC clinical practice guideline for prescribing opioids for pain — United states, 2022. MMWR Recomm Rep. (2022) 71:1–95. doi: 10.15585/mmwr.rr7103a1, PMID: 36327391 PMC9639433

[7] El Hayek S Geagea L El Bourji H Kadi T Talih F . Prevention strategies of alcohol and substance use disorders in older adults. Clin Geriatr Med. (2022) 38:169–79. doi: 10.1016/j.cger.2021.07.011, PMID: 34794700

[8] Carroll KM . The profound heterogeneity of substance use disorders: Implications for treatment development. Curr Dir Psychol Sci. (2021) 30:358–64. doi: 10.1177/09637214211026984, PMID: 34483503 PMC8415637

[9] Farhoudian A Razaghi E Hooshyari Z Noroozi A Pilevari A Mokri A . Barriers and facilitators to substance use disorder treatment: An overview of systematic reviews. Subst abuse: Res Treat. (2022) 16:11782218221118462. doi: 10.1177/11782218221118462, PMID: 36062252 PMC9434658

[10] Caris L Beckers T . Accessibility of substance use treatment: A qualitative study from the non-service users’ perspective. J Subst Abuse Treat. (2022) 141:108779. doi: 10.1016/j.jsat.2022.108779, PMID: 35864014

[11] Krishnamurthy N Grimshaw AA Axson SA Choe SH Miller JE . Drug repurposing: a systematic review on root causes, barriers and facilitators. BMC Health Serv Res. (2022) 22:970. doi: 10.1186/s12913-022-08272-z, PMID: 35906687 PMC9336118

[12] Nauck MA Quast DR Wefers J Meier JJ . GLP-1 receptor agonists in the treatment of type 2 diabetes–state-of-the-art. Mol Metab. (2021) 46:101102. doi: 10.1016/j.molmet.2020.101102, PMID: 33068776 PMC8085572

[13] Bruns Vi N Tressler EH Vendruscolo LF Leggio L Farokhnia M . IUPHAR review - Glucagon-like peptide-1 (GLP-1) and substance use disorders: An emerging pharmacotherapeutic target. Pharmacol Res. (2024) 207:107312. doi: 10.1016/j.phrs.2024.107312, PMID: 39032839 PMC11467891

[14] Farr OM Sofopoulos M Tsoukas MA Dincer F Thakkar B Sahin-Efe A . GLP-1 receptors exist in the parietal cortex, hypothalamus and medulla of human brains and the GLP-1 analogue liraglutide alters brain activity related to highly desirable food cues in individuals with diabetes: a crossover, randomised, placebo-controlled trial. Diabetologia. (2016) 59:954–65. doi: 10.1007/s00125-016-3874-y, PMID: 26831302 PMC4826792

[15] Psilopanagioti A Nikou S Logotheti S Arbi M Chartoumpekis DV Papadaki H . Glucagon-like peptide-1 receptor in the human hypothalamus is associated with body mass index and colocalizes with the anorexigenic neuropeptide nucleobindin-2/nesfatin-1. Int J Mol Sci. (2022) 23:14899. doi: 10.3390/ijms232314899, PMID: 36499229 PMC9740138

[16] Brunchmann A Thomsen M Fink-Jensen A . The effect of glucagon-like peptide-1 (GLP-1) receptor agonists on substance use disorder (SUD)-related behavioural effects of drugs and alcohol: a systematic review. Physiol behavior. (2019) 206:232–42. doi: 10.1016/j.physbeh.2019.03.029, PMID: 30946836 PMC6520118

[17] Jensen ME Galli A Thomsen M Jensen KL Thomsen GK Klausen MK . Glucagon-like peptide-1 receptor regulation of basal dopamine transporter activity is species-dependent. Neurochemistry Int. (2020) 138:104772. doi: 10.1016/j.neuint.2020.104772, PMID: 32464226 PMC7452124

[18] Vallof D Maccioni P Colombo G Mandrapa M Jornulf JW Egecioglu E . The glucagon-like peptide 1 receptor agonist liraglutide attenuates the reinforcing properties of alcohol in rodents. Addict Biol. (2016) 21:422–37. doi: 10.1111/adb.12295, PMID: 26303264 PMC5049632

[19] Lahteenvuo M Tiihonen J Solismaa A Tanskanen A Mittendorfer-Rutz E Taipale H . Repurposing semaglutide and liraglutide for alcohol use disorder. JAMA Psychiatry. (2025) 82:94–8. doi: 10.1001/jamapsychiatry.2024.3599, PMID: 39535805 PMC11561716

[20] Yammine L Verrico CD Versace F Webber HE Suchting R Weaver MF . Exenatide as an adjunct to nicotine patch for smoking cessation and prevention of postcessation weight gain among treatment-seeking smokers with pre-diabetes and/or overweight: study protocol for a randomised, placebo-controlled clinical trial. BMJ Open. (2023) 13:e072707. doi: 10.1136/bmjopen-2023-072707, PMID: 37316311 PMC10277057

[21] Probst L Monnerat S Vogt DR Lengsfeld S Burkard T Meienberg A . Effects of dulaglutide on alcohol consumption during smoking cessation. JCI Insight. (2023) 8:e170419. doi: 10.1172/jci.insight.170419, PMID: 37991022 PMC10721313

[22] Lengsfeld S Burkard T Meienberg A Jeanloz N Vukajlovic T Bologna K . Effect of dulaglutide in promoting abstinence during smoking cessation: a single-centre, randomized, double-blind, placebo-controlled, parallel group trial. EClinicalMedicine. (2023) 57:101865. doi: 10.1016/j.eclinm.2023.101865, PMID: 36874396 PMC9981899

[23] Klausen MK Thomsen M Wortwein G Fink-Jensen A . The role of glucagon-like peptide 1 (GLP-1) in addictive disorders. Br J Pharmacol. (2022) 179:625–41. doi: 10.1111/bph.15677, PMID: 34532853 PMC8820218

[24] Angarita GA Matuskey D Pittman B Costeines JL Potenza MN Jastreboff AM . Testing the effects of the GLP-1 receptor agonist exenatide on cocaine self-administration and subjective responses in humans with cocaine use disorder. Drug Alcohol Depend. (2021) 221:108614. doi: 10.1016/j.drugalcdep.2021.108614, PMID: 33621809 PMC8026565

[25] Investigators AOURP . The “All of Us” research program. New Engl J Med. (2019) 381:668–76. doi: 10.1056/NEJMsr1809937, PMID: 31412182 PMC8291101

[26] Amorim Moreira Alves G Teranishi M Teixeira de Castro Gonçalves Ortega AC James F Perera Molligoda Arachchige AS . Mechanisms of GLP-1 in modulating craving and addiction: neurobiological and translational insights. Med Sci. (2025) 13:136. doi: 10.3390/medsci13030136, PMID: 40843757 PMC12372146

[27] Marquez-Meneses JD Olaya-Bonilla SA Barrera-Carreño S Tibaduiza-Arévalo LC Forero-Cárdenas S Carrillo-Vaca L . GLP-1 analogues in the neurobiology of addiction: translational insights and therapeutic perspectives. Int J Mol Sci. (2025) 26:5338. doi: 10.3390/ijms26115338, PMID: 40508146 PMC12155186

[28] Von Elm E Altman DG Egger M Pocock SJ Gøtzsche PC Vandenbroucke JP . The Strengthening the Reporting of Observational Studies in Epidemiology (STROBE) statement: guidelines for reporting observational studies. Lancet. (2007) 370:1453–7. doi: 10.1016/S0140-6736(07)61602-X, PMID: 18064739

[29] Gleason PP Urick BY Marshall LZ Friedlander N Qiu Y Leslie RS . Real-world persistence and adherence to glucagon-like peptide-1 receptor agonists among obese commercially insured adults without diabetes. J Managed Care Specialty Pharmacy. (2024) 30:860–7. doi: 10.18553/jmcp.2024.23332, PMID: 38717042 PMC11293763

[30] Wallach JD O’Malley SS Lipska KJ Ross JS Jeffery MM Savitz ST . Trends in newly filled GLP-1 receptor agonist prescriptions for US patients with versus without comorbid alcohol use disorder, 2016–2024. J Addict Med. (2024) 10:1097. doi: 10.1097/ADM.0000000000001575, PMID: 41036780 PMC12636227

[31] Liu QK . Mechanisms of action and therapeutic applications of GLP-1 and dual GIP/GLP-1 receptor agonists. Front Endocrinology. (2024) 15:1431292. doi: 10.3389/fendo.2024.1431292, PMID: 39114288 PMC11304055

[32] Hernandez N Schmidt H . Central GLP-1 receptors: Novel molecular targets for cocaine use disorder. Physiol behavior. (2019) 206:93–105. doi: 10.1016/j.physbeh.2019.03.026, PMID: 30930091 PMC6520198

[33] Bucciarelli L Cimino V Dell’Osso B Fiorina P . Psychotropic effects of GLP-1R agonists. Pharmacol Res. (2025) 108036. doi: 10.1016/j.phrs.2025.108036, PMID: 41253199

[34] Skibicka KP . The central GLP-1: implications for food and drug reward. Front Neurosci. (2013) 7:181. doi: 10.3389/fnins.2013.00181, PMID: 24133407 PMC3796262

[35] Hendershot CS Bremmer MP Paladino MB Kostantinis G Gilmore TA Sullivan NR . Once-weekly semaglutide in adults with alcohol use disorder: A randomized clinical trial. JAMA Psychiatry. (2025) doi: 10.1001/jamapsychiatry.2024.4789, PMID: 39937469 PMC11822619

