# Supplementary material for: Association between GLP-1 receptor agonist use and substance use disorders among individuals with type 2 diabetes or obesity: a nested case-control study in the All of Us research program
Source: Front Psychiatry. 2026 Mar 10;17:1766770. doi: 10.3389/fpsyt.2026.1766770 (PMC13008929; doi:10.3389/fpsyt.2026.1766770)
Supplement: Supplementary file 1 [file Supplementaryfile1.docx]

Supplementary Material

# Supplementary Figures and Tables

## Supplementary Tables

Supplementary Table 1 provides detailed numerical estimates corresponding to Figures 1–5 in the main manuscript. The table reports adjusted odds ratios (AORs), 95% confidence intervals (CIs), and p-values for the association between exposure to glucagon-like peptide-1 receptor agonists (GLP-1RAs)—both overall exposure and individual agents (dulaglutide, exenatide, liraglutide, and semaglutide)—and the odds of alcohol use disorder (AUD), opioid use disorder (OUD), nicotine use disorder (NUD), cocaine use disorder (CUD), and any substance use disorder (any SUD). This supplementary table is intended to provide complete numerical results underlying the forest plots presented in the main text and figures, enabling transparency, and precise interpretation of effect sizes and statistical significance across substance use disorder outcomes.

Supplementary Table 1. Adjusted odds ratios for substance use disorders associated with GLP-1 receptor agonist exposure in the All of Us Cohort

| Medications | Type of SUD | | | | | | | | | |
| --- | --- | --- | --- | --- | --- | --- | --- | --- | --- | --- |
|  | AUD | | OUD | | NUD | | CUD | | Any type of SUD | |
|  | OR [95%CI] | p-value | OR [95%CI] | p-value | OR [95%CI] | p-value | OR [95%CI] | p-value | OR [95%CI] | p-value |
| Any GLP-1 exposure | 0.26[0.20-0.34] | <0.01 | 0.31[0.23-0.42] | <0.01 | 0.32[0.27–0.39] | <0.01 | 0.25[0.16-0.40] | <0.01 | 0.25[0.22–0.30] | <0.01 |
| Dulaglutide | 0.18[0.10-0.33] | <0.01 | 0.36[0.20-0.64 | <0.01 | 0.31[0.21-0.44] | <0.01 | 0.26[0.11-0.60] | <0.01 | 0.22[0.16–0.31] | <0.01 |
| Exenatide | 0.25[0.13-0.48] | <0.01 | 0.28[0.13-0.59] | <0.01 | 0.30[0.19-0.47] | <0.01 | 0.56[0.19-1.66] | 0.29 | 0.26[0.18–0.37] | <0.01 |
| Liraglutide | 0.27[0.18-0.40] | <0.01 | 0.29[0.19-0.46] | <0.01 | 0.30[0.23-0.40] | <0.01 | 0.13[0.05-0.32] | <0.01 | 0.23[0.18–0.29] | <0.01 |
| Semaglutide | 0.15[0.07-0.34] | <0.01 | 0.13[0.04-0.43] | <0.01 | 0.38[0.24-0.59] | <0.01 | 0.27[0.08-0.98] | <0.01 | 0.30[0.20–0.44] | <0.01 |

- 1. Supplementary Figures


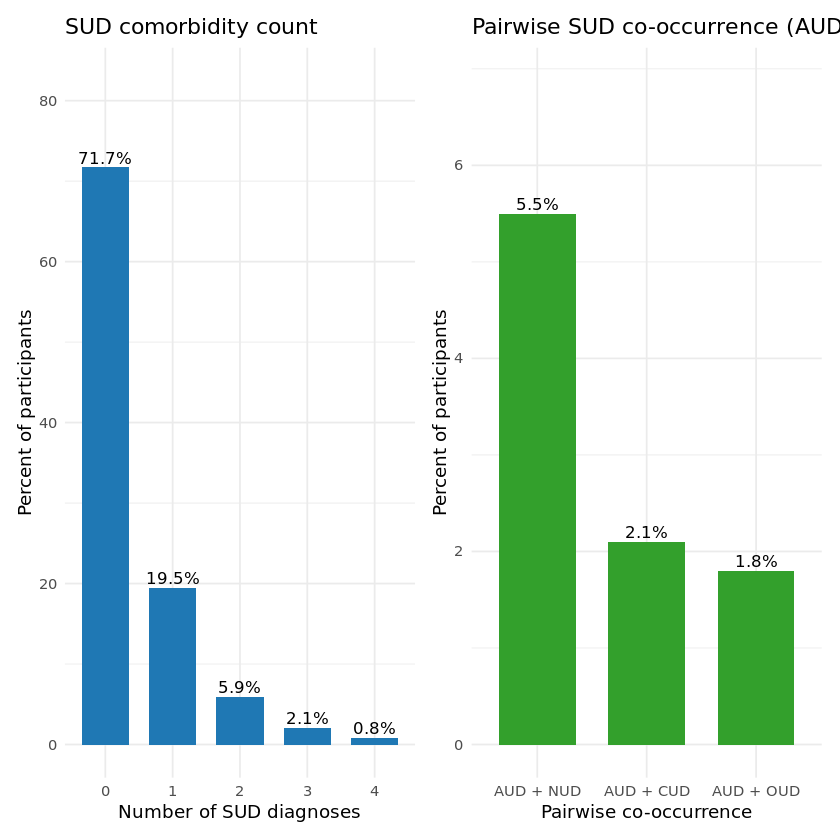


Supplementary Figure1: Distribution and pairwise co-occurrence of substance use disorders (SUDs) in the All of Us cohort.

**Legend:** In Supplementary Figure1, the left panel (blue bars) displays the distribution of the number of substances use disorder per participant, ranging from zero to four, across AUD, OUD, NUD, and CUD. The right panel (green bars) shows the percentage of participants with pairwise co-occurrence of AUD with OUD, NUD, or CUD. Percentages are calculated using the total study population as the denominator. Values above bars indicate the proportion of participants in each category. AUD, alcohol use disorder; OUD, opioid use disorder; NUD, nicotine use disorder; CUD, cocaine use disorder.


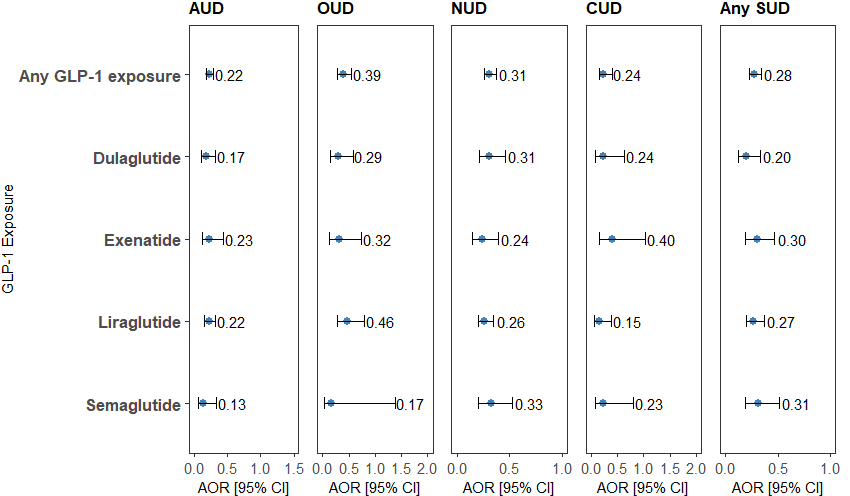


Supplementary Figure2: The association between GLP-1 RA exposure and the odds of substance use disorders in females.


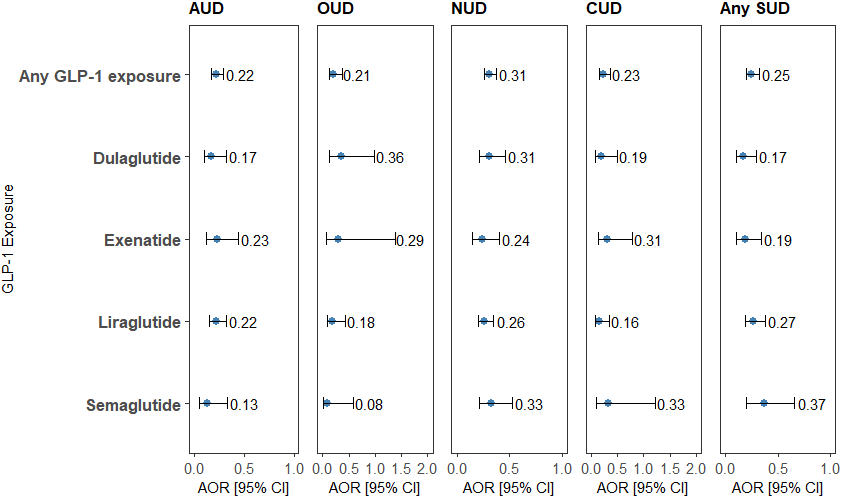


Supplementary Figure3: The association between GLP-1 RA exposure and the odds of substance use disorders in males.

**Legend:** Supplementary Figures 2 and 3 illustrate the association between GLP-1 receptor agonist exposure and the odds of substance use disorders stratified by sex. Supplementary Figure 2 presents adjusted odds ratios (AORs) for females, and Supplementary Figure 3 presents corresponding estimates for males. Across all SUD outcomes (AUD, OUD, NUD, CUD, and any SUD) and individual GLP-1 RA agents, the direction and magnitude of associations were generally similar between females and males. Overall, subgroup analyses by sex did not indicate meaningful differences in the association between GLP-1 RA exposure and the odds of substance use disorders, suggesting no evidence of effect modification by sex. AUD, alcohol use disorder; OUD, opioid use disorder; NUD, nicotine use disorder; CUD, cocaine use disorder.


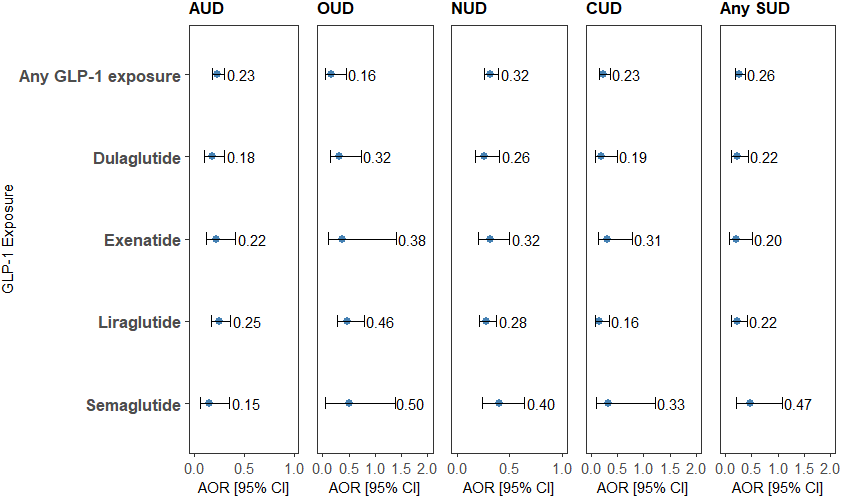


Supplementary Figure4: The association between GLP-1 RA exposure and the odds of substance use disorders in diabetes patients without obesity.


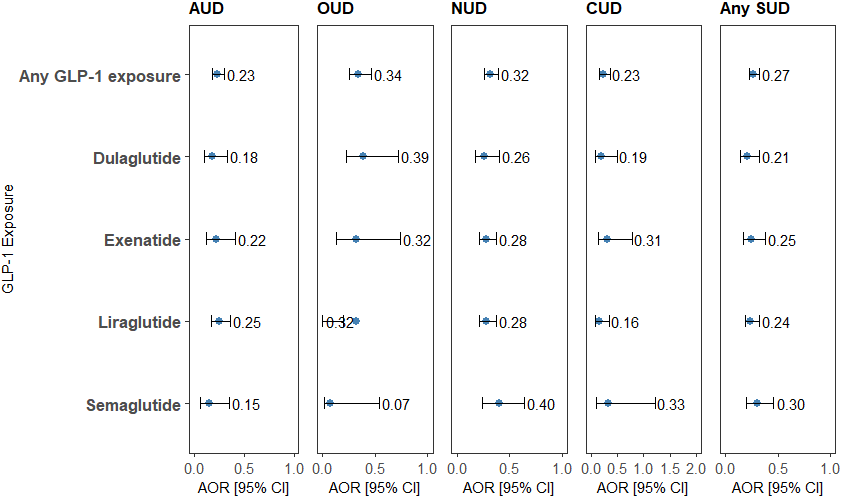


Supplementary Figure5: The association between GLP-1 RA exposure and the odds of substance use disorders in diabetes patients with obesity.

**Legend:** Supplementary Figures 4 and 5 show the association between GLP-1 receptor agonist (GLP-1 RA) exposure and the odds of substance use disorders stratified by obesity status among patients with diabetes. Supplementary Figure 4 presents adjusted odds ratios (AORs) for patients without obesity, while Supplementary Figure 5 presents corresponding estimates for patients with obesity. Across all SUD outcomes (AUD, OUD, NUD, CUD, and any SUD) and individual GLP-1 RA agents, the direction and magnitude of associations were generally similar between patients with and without obesity. Overall, the subgroup analyses by obesity status did not indicate meaningful differences in the association between GLP-1 RA exposure and the odds of substance use disorders, suggesting no evidence of effect modification by obesity status. AUD, alcohol use disorder; OUD, opioid use disorder; NUD, nicotine use disorder; CUD, cocaine use disorder.
